# Epigenetic and polygenic contributions to body mass index: a validation study of predictive models

**DOI:** 10.1186/s13148-025-02040-6

**Published:** 2026-03-06

**Authors:** Kamila Marszałek, Maria Wróbel, Balakrishnan Subramanian, Krystyna Jaworska, Aleksandra Pisarek-Pacek, Danuta Piniewska-Róg, Karol Makiel, Agnieszka Suder, Małgorzata Michalczyk, Bożena Wysocka, Aneta Sitek, Magdalena Spólnicka, Andrzej Ossowski, Wojciech Branicki, Ewelina Pośpiech

**Affiliations:** 1 Department of Genomics and Forensic Genetics, Szczecin, Pomeranian Medical University in Szczecin, Szczecin, Poland; 2 Institute of Zoology and Biomedical Research, Jagiellonian University, Krakow, Poland; 3 Institute of Forensic Research, Krakow, Poland; 4 Faculty of Medicine, Department of Forensic Medicine, Jagiellonian University Medical College, Krakow, Poland; 5 Faculty of Physical Rehabilitation, Department of Anatomy, University of Physical Culture in Krakow, Krakow, Poland; 6 The Jerzy Kukuczka Academy of Physical Education in Katowice, Institute of Sport Science, Katowice, Poland; 7 Medict Institute, Gliwice, Poland; 8 Central Forensic Laboratory of the Police, Warsaw, Poland; 9 Faculty of Biology and Environmental Protection, Department of Anthropology, University of Lodz, Lodz, Poland; 10 Center for Forensic Science, University of Warsaw, Warsaw, Poland; 11 Regional Center of Digital Medicine, Pomeranian Medical University in Szczecin, Szczecin, Poland

**Keywords:** Body mass index, BMI prediction, Epigenetic profiling, Polygenic risk score, Metabolic health, Forensic DNA phenotyping

## Abstract

Body mass index (BMI) has a notable genetic component, yet the majority of genetic variants remain elusive. Their identification may be hindered as weight also stems from exogenous factors and may be shaped by lifestyle influences. Since DNA methylation is partially controlled by genes and also captures environmental exposures, epigenetic biomarkers have been successfully employed in BMI prediction. In this study, we integrated SNP and DNA methylation data to independently validate existing epigenetic models and polygenic risk scores for predicting BMI. In a sample of 624 adult Polish individuals, the top ten CpG sites accounted for nearly twice the variance in phenotypic BMI compared to the top ten SNPs. The polygenic risk score formulated by Yengo et al. demonstrated a highly significant, low-to-moderate correlation with BMI (*r* = 0.249, *p* = 2.7 × 10^− 10^). Four published epigenetic predictors, trained on up to 2,000 CpG sites, were evaluated in our sample, with a significant moderate correlation with BMI observed across all models (*r* = 0.4–0.5). Next, predicted values were transformed onto the absolute BMI scale and applied to an independent validation set (*N* = 112). The mean absolute error (MAE) of BMI prediction was comparable across all models, averaging ± 3.1 units, with the Smith model achieving the highest predictive accuracy (MAE = 2.7). Notably, the predictor developed by Do et al. showed the most consistent association between the deviation in epigenetic versus phenotypic BMI and multiple health-related epigenetic biomarkers. The strongest association was observed for body fat percentage (β > 0.6), while moderate effect sizes were found for waist-to-hip ratio, alcohol consumption, epigenetic CRP, HDL cholesterol, and smoking. This study provides new insights into the relative roles of genetic and epigenetic markers in BMI prediction. While known SNPs contribute to the explained variance, CpG markers exhibited stronger effect sizes in the studied population. The BMI values returned by the models showed associations with multiple epigenetic health indicators, underscoring their potential translational value for metabolic health assessment. This study can also inform the selection of BMI predictors suitable for forensic genetic analyses, supporting the reconstruction of an individual’s appearance from secured biological evidence.

**Supplementary Information:**

The online version contains supplementary material available at 10.1186/s13148-025-02040-6.

## Background

Obesity is a global health concern, and the burden it places on healthcare systems has increased by more than 10% in recent years [1]. Body mass index (BMI), calculated as body weight divided by height squared, remains a central clinical measure of obesity [2–4]. Although BMI is widely utilized, it presents notable limitations as an indicator of metabolic health. For instance, it does not account for the amount or distribution of body fat [5, 6], nor can it distinguish between metabolically healthy individuals and those with metabolic dysfunction who fall within the normal weight range [7]. Emerging research suggests that epigenetically predicted BMI could offer greater accuracy than phenotypic BMI, derived solely from weight and height [3, 6], as it more effectively reflects the complex biological mechanisms underlying metabolic health beyond obesity [5].

While genetic predisposition is estimated to account for approximately 40–60% of the variability in BMI [8, 9], common genetic variants and corresponding polygenic scores explain only a modest proportion, around 5% to 10%, of this variation [10, 11]. Importantly, DNA methylation has been shown to contribute additional, independent explanatory power, raising the total proportion of explained variance to between 13% and 20% [10, 11]. Methylation patterns are 20–40% under genetic control [12, 13]. However, part of DNA methylation is dynamic and can be influenced by various exogenous factors, playing a role of a mediator between the genetics and the environment. DNA methylation at numerous CpG sites has been associated with BMI, with over 3,700 sites reported across 11 different studies [5, 10, 11, 14–21]. Subsequently, several epigenetic scores for BMI were developed, each trained on a substantial number of CpG sites, ranging from 400 to 2,000 [5, 11, 14, 15]. These models have demonstrated applicability across diverse populations [5], although ethnicity-related interactions have been reported for BMI associations at specific CpG sites [15]. Notably, epigenetic scores for BMI have been associated in the literature with a range of health-related outcomes, including metabolic syndrome, triglyceride levels, cognitive function, lung function, physical health, and quality of life [5, 14, 15]. Integrating SNP and DNA methylation data presents a promising approach to further enhance the accuracy and predictive power of BMI estimation [10]. One study investigated the combined predictive power of these two data types, reporting a moderate ability to distinguish between obese and non-obese individuals. The area under the ROC curve (AUC) was 0.67 when using DNA methylation data alone, and increased to 0.71 when a genetic score was included [11].

Importantly, BMI prediction based on (epi)genetic data can also hold relevance for other applied fields, including forensic genetics and anthropology [22]. Forensic DNA phenotyping is an emerging field that focuses on developing DNA-based solutions to support the investigative phase of criminal cases. The goal is to extract as much information as possible from DNA to aid in suspect profiling when other leads are lacking [23, 24]. Assessing the appearance of the individual who provided the biological material, as well as evaluating biogeographical ancestry and age, can help guide investigations and identification efforts. Importantly, the ability to infer BMI or obesity status could complement efforts to reconstruct the appearance of a criminal suspect based on biological material recovered from a crime scene or to aid in the identification of an individual from human remains. In particular, BMI prediction could improve the accuracy of facial phenotype reconstruction [25, 26].

In the present study, we conducted a comprehensive validation and comparison of four established epigenetic BMI prediction models and two polygenic risk scores. The comparison included the set and number of markers, the strength of correlation with BMI, the BMI prediction error, and associations with other health-related metrics. Such a comparison may have important clinical relevance, as it can guide the selection of appropriate methods in specific studies and for potential future implementation. Additionally, comparing the BMI prediction error across tools may help inform the choice of optimal methods in forensic applications. The analyses were performed using microarray-derived DNA methylation and SNP data obtained from a total of 736 blood samples collected from adult individuals of Polish origin. The predictive performance of each model for BMI was assessed, and the correlation between BMI prediction error and a set of epigenetic health biomarkers, including body fat percentage, smoking status, HDL cholesterol, and CRP levels, was evaluated. Our findings contribute to a deeper understanding of the respective roles of genetic and epigenetic variation in BMI prediction and offer novel insights into the biological significance of epigenetically derived BMI measures in the context of metabolic and inflammatory health phenotypes.

## Materials and methods

### Study cohort and phenotypic measurements

The cohort under study has been previously described [27]. BMI data (mean BMI = 26.4, SD = 4.4), excluding individuals with extreme BMI values (BMI of > 45 kg/m2) were available for 736 unrelated individuals from Poland aged between 18 and 87 years (mean age = 46.1, SD = 14.8, Table S1). Blood samples and lifestyle questionnaires were collected from all study participants [27]. Weight (kg) and height (cm) were measured using a digital scale and a stadiometer, respectively, according to a standardized protocol. Phenotypic BMI was determined as weight in kilograms divided by height in meters squared (kg/m²). For the purpose of association testing, BMI was corrected for age and sex in linear regression analysis, and the resulting residuals were then subjected to inverse normal transformation to match a standard normal distribution (Fig. S1).

The cohort was randomly divided into two sets, Set 1 (*N* = 624, 85%) and Set 2 (*N* = 112, 15%), while maintaining equal distributions of age, sex, and BMI across both groups. The larger set served as the main dataset and was used to validate existing genetic and epigenetic predictors of BMI. Moreover, because available epigenetic predictors return values on a relative rather than an absolute BMI scale, the main dataset was also used to map predicted values onto actual phenotypic BMI values. To this end, a linear regression analysis was performed, with phenotypic BMI treated as the independent variable and the BMI prediction outcome from the models as the dependent variable. The resulting transformation equation, derived from the main dataset (Set 1), was then applied to an independent smaller set of samples (Set 2), which was used to evaluate the calibrated BMI predictions and compare the models.

Ethical permission was obtained from the Bioethics Committee of the Jagiellonian University in Krakow (decisions no. 1072.6120.132.2018, 1072.6120.31.2025) and written informed consent was obtained from all participants.

### Genomic and epigenomic data collection

Whole-blood samples were subjected to DNA extraction using an automated method with the Maxwell RSC Blood DNA Kit. The extracted DNA was then profiled for genome-wide DNA methylation and SNPs using the Illumina Infinium Methylation EPIC microarray and the Global Screening Array (v3), respectively, as described elsewhere [27]. Quantile normalization was applied to the methylation data, and beta values (the ratio of methylated probe intensity to total probe intensity) were extracted for the target CpG list. For certain analyses, as indicated in the relevant sections of Materials and Methods, scaled beta values were used. Genotypic data for the target SNP polymorphism list were also extracted, with genotypes encoded as 0, 1, and 2, following the additive model.

### SNP association and polygenic risk score analysis

To assess population structure within the cohort and to generate ancestry covariates for association analyses, principal component analysis (PCA) [28] was conducted on high-quality, LD-pruned SNPs using PLINK v1.9 [29]. The following variant-level filters were applied: minor allele frequency (MAF > 0.05), genotype missingness (GENO < 0.01), and retention of biallelic SNPs only. Linkage disequilibrium (LD) pruning was performed using a 200-SNP window, a 50-SNP step size, and an r² threshold of 0.1.

Eight studies were retrieved from the literature on BMI-associated SNPs [30–37]. In total, 1,450 SNPs were initially pre-selected; however, only 371 of these were present on the Infinium GSA v3 array (**Table S2**). These 371 SNPs were extracted for a study population of *N* = 624 and subjected to association testing with inverse-normalized BMI residuals, using linear regression adjusted for smoking status, age and 10 top Principal Components (PCs).

Next, the largest meta-analysis of genome-wide association studies (GWAS) to date, conducted by Yengo et al. in approximately 700,000 individuals of European ancestry [32], was used to calculate the polygenic risk score (PRS; the sum of effect allele counts weighted by their effects) in our study population (*N* = 624), based on 941 reported SNPs. GWAS statistics were retrieved from the GIANT consortium website. Polygenic risk scores were computed using PRS-CS, a Bayesian regression framework that applies continuous shrinkage priors and incorporates external linkage disequilibrium (LD) structure to improve cross-population portability [38]. Prior to PRS construction, genotype data underwent standard QC using PLINK (SNP missingness threshold: GENO < 0.05, individual missingness: MIND < 0.05, Hardy–Weinberg equilibrium: *p* > 1 × 10⁻⁶, minor allele frequency: MAF > 0.01, retention of biallelic ACGT variants only). PRS-CS was executed separately for autosomes (chromosomes 1–22) using the 1,000 Genomes European LD reference panel (ldblk_1kg_eur) [39]. Default parameters were used, including automatic global shrinkage (phi = auto). Posterior effect sizes for each chromosome were merged into a unified weight file. Individual-level PRSs were computed in PLINK 1.9. These PRS values were standardized (Z-scores) before downstream association analyses.

Additionally, another PRS (PGS001825) from the PGS Catalog [40, 41], developed for overweight status, was applied to our dataset. This score includes 13,009 genome-wide SNPs with reported effect alleles and β-weights derived from penalized regression (PLR) models. PRS values were computed in PLINK, after which both the PRS-CS–derived BMI scores and the overweight-status PGS were analyzed for distribution and density, correlation with phenotypic BMI (inverse-normalized BMI residuals), quantile effects (Q1–Q5), sex differences, and BMI-category differences.

### Validation of established epigenetic predictors of BMI

A literature data search was conducted to pick out BMI-associated CpG sites and to screen for available epigenetic predictors of BMI. In total, eleven studies were considered [5, 10, 11, 14–21], yielding a target list of 3,721 unique BMI-linked CpG sites present on the EPIC microarray (**Table S3**), which were subsequently subjected to association testing in our study population (*N* = 624). In linear regression analyses, inverse-normalized BMI residuals, after regressing on age and sex, were used as the outcome variable, while beta methylation values for each CpG probe were included as the independent variable, with additional adjustment for smoking status, age and 10 top Principal Components (PCs). Principal component analysis (PCA) was performed on the methylation data using the prcomp() function in R to capture major sources of variation across samples. Finally, the Yengo et al. PRS score [32] was also included as a covariate in regression analyses to condition the association between candidate CpG methylation and BMI on genetic variation as part of a sensitivity analysis.

**Fig. 1:**
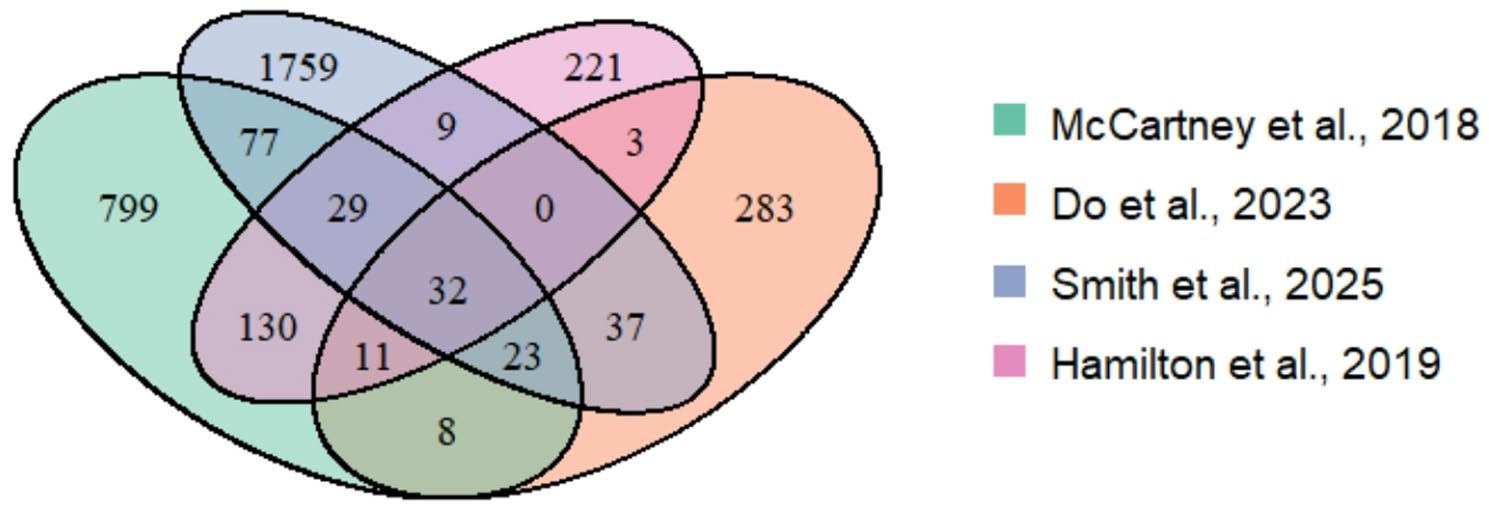
Venn diagram showing the overlap of CpGs analyzed by the four epigenetic BMI predictors

A review of the relevant literature identified four studies that reported epigenetic predictors together with their CpG weights [5, 11, 14, 15]. Based on these published predictors, BMI epigenetics scores were subsequently computed following the procedures outlined in the respective studies. Beta methylation values, following quantile normalization [42], were used as input and multiplied by the weights of the corresponding CpG probes, then summed, with the intercept added when available. For one of the BMI models [5], the beta values were standardized beforehand, that is, scaled to a mean of 0 and a variance of 1. The earliest study, by McCartney et al. in 2018, analyzes 1,109 CpG sites in the genome [11]. The subsequent study, by Hamilton et al. in 2019, examines 435 CpG markers [14], while the paper published by Do et al. in 2023 investigates 397 CpG sites [15]. The most recent study, from 2025, estimates BMI based on the analysis of 1,966 CpG sites in the genome [5]. The overlap in CpG content among the BMI predictors is illustrated in a Venn diagram (Fig. 1).

Since the BMI predictor developed by Do et al. [15] was trained using data from a previous version of the array, namely the Infinium 450 K, 28 out of 397 CpG sites were missing on the EPIC array and were imputed using the mean value approach. Mean values for the corresponding CpG probes were extracted from the GSE56105 dataset [43]. In the case of the McCartney et al. model, the MethylDetectR tool was used to derive BMI scores, with automatic data imputation applied if needed [44].

Importantly, in all four epigenetic predictors, BMI was standardized or regressed on age, sex, and other confounders prior to model training. Therefore, the outputs of these models are not absolute BMI values and can only be interpreted on a relative scale. The BMI scores from all predictors were analyzed for correlation with phenotypic BMI (inverse-normalized BMI residuals), and the resulting Pearson correlation coefficients were compared. Next, to evaluate BMI prediction error, linear regression was used to determine the correlation between the epigenetic scores from the respective models and phenotypic BMI in our Set 1, consisting of *N* = 624 samples. This type of transformation was then applied to an independent test set (Set 2, *N* = 112) to assess prediction accuracy on the original BMI scale.

### Association with other epigenetic metabolic scores

Epigenetic proxies for other measures of metabolic health or inflammation were derived using published tools. Body fat percentage, HDL cholesterol level, waist-to-hip ratio, alcohol consumption, and smoking scores were calculated with the MethylDetectR tool, based on a total of over 3,000 CpGs, as described in the original study by Marioni et al. [44]. Epigenetic CRP, on the other hand, was computed using beta coefficient–based weights for six (cg06690548, cg10636246, cg18181703, cg19821297, cg25325512, cg27023597) of the seven CpGs reported by Ligthart et al. [45, 46]; one CpG (cg06126421) was missing in the EPIC microarrays and therefore excluded from the calculation. Beta methylation values were used as input for these analyses, consistent with the procedures described in the original studies.

To evaluate whether discrepancies between epigenetic and phenotypic BMI have potential biological relevance, residuals were calculated from a regression of epigenetic BMI on phenotypic BMI, adjusted for age and sex. These residuals capture the component of epigenetic BMI not explained by phenotypic BMI. Subsequent linear regression analyses assessed associations between the BMI residuals and the epigenetic metabolic scores, additionally controlling for phenotypic BMI.

## Results

### Genetic variants and DNA methylation sites associated with BMI

**Fig. 2:**
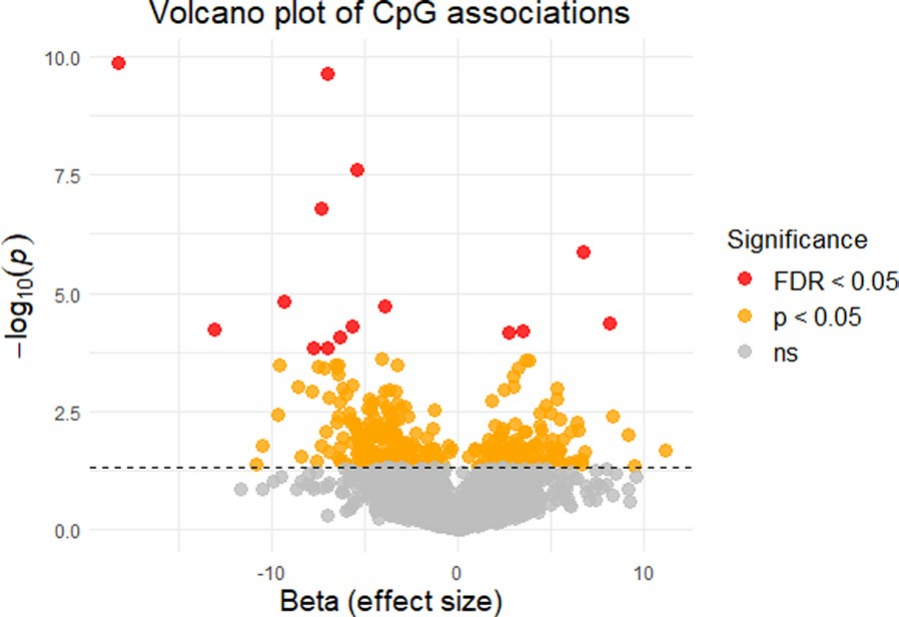
Volcano plot of differentially methylated CpGs associated with phenotypic BMI. Analysis was performed on literature-reported CpGs (*N * = 3,721) in a sample set of 624 Polish subjects, using linear regression with additional adjustment for smoking status age, and the first 10 Principal Components (PCs). Phenotypic BMI, expressed as inverse-normalized residuals after regressing on age and sex, was used as the dependent variable in the association tests.

SNP and CpG sites identified in prior studies were tested for association with BMI in a sample of 624 adult Polish subjects (Set 1). Nominal significance (*p* < 0.05) was detected at 18 of 371 SNP positions (4.9%) and 320 of 3,721 CpG loci (8.6%) (**Table S2 and S3**, respectively). No SNPs passed FDR correction, but the top loci included rs11688816 located in the *EHBP1* gene (raw *p* = 0.0007) and rs4776970 in the *MAP2K5* gene (raw *p* = 0.003, Table 1). Minor alleles were linked to higher BMI in 55% of nominally significant SNPs. In contrast, 15 differentially methylated positions reached FDR-corrected significance (Fig. 2), with cg00574958 at *CPT1A* (raw *p* = 1.4 × 10^− 10^) and cg17901584 at *DHCR24* (raw *p* = 2.3 × 10^− 10^) emerging as the top two markers (Table 2). Lower methylation values were usually linked to higher BMI; 190 (59.4%) nominally significant CpGs exhibited hypomethylation corresponding to BMI increase.

### Explained variance in BMI: CpGs and SNPs

The observed change in BMI was, in principle, more substantial per 10% increase in methylation beta values than per allele increase in the case of SNPs. The methylation range, defined as the difference between the maximum and minimum beta values observed across the population, had a mean of 0.25 ± 0.12. The vast majority of SNPs with nominally significant associations were common, having a minor allele frequency (MAF) greater than 15%, only one SNP was low frequency (MAF < 5%, rs118067556). The top ten CpGs accounted for almost twice as much variance in BMI as the top ten SNPs, with adjusted R² values of 15.5% and 8.2%, respectively (Tables 1 and 2).

**Table 1:** Top ten SNP positions associated with BMI in a sample of 624 Polish subjects

Top SNP associations												
No.	SNP ID	Chr	Chr position (hg19)	Gene	A1	A2	MAF	beta	SE	*p*	FDR *p*	BMI change
1	rs11688816	2	63,053,048	*EHBP1*	A	G	0.496	-0.195	0.057	0.0007	0.263	-0.778 [-1.261 – -0.295]
2	rs4776970	15	68,080,886	*MAP2K5*	T	A	0.361	0.182	0.060	0.003	0.483	0.655 [0.150–1.161]
3	rs10733682	9	129,460,914	*LMX1B*	G	A	0.464	-0.154	0.056	0.006	0.738	-0.669 [-1.138 – -0.201]
4	rs10968576	9	28,414,339	*LINGO2*	G	A	0.355	-0.152	0.060	0.011	0.782	-0.725 [-1.225 – -0.226]
5	rs7164727	15	73,093,991	NA	C	T	0.320	0.156	0.063	0.014	0.782	0.600 [0.067–1.133]
6	rs2176040	2	227,092,802	NA	A	G	0.381	-0.141	0.058	0.015	0.782	-0.484 [-0.971–0.002]
7	rs10754210	1	197,012,111	*F13B*	A	G	0.302	0.154	0.063	0.015	0.782	0.634 [0.102–1.166]
8	rs118067556	10	63,136,165	NA	T	C	0.018	0.505	0.218	0.021	0.782	1.655 [-0.178–3.487]
9	rs7141420	14	79,899,454	*NRXN3*	C	T	0.402	-0.136	0.059	0.022	0.782	-0.544 [-1.041 – -0.047]
10	rs9400239	6	108,977,663	*FOXO3*	T	C	0.345	0.135	0.060	0.026	0.782	0.583 [0.077–1.089]

BMI, measured as inverse-normal BMI residuals after regressing on age and sex, was treated as the outcome, with individual SNPs as independent variables in the linear regression analysis. The results were additionally adjusted for smoking status, age, and the first ten Principal Components (PCs). BMI change is defined as the change in raw BMI (kg/m²) per additional effect allele, with confidence intervals CI provided in brackets. MAF for allele A1.

**Table 2:** Top ten CpG sites associated with BMI in a sample of 624 Polish subjects

Top CpG associations										
No.	CpG ID	Chr	Chr position (hg19)	Gene	beta	SE	Mean methyl. ±SD	*p*	FDR *p*	BMI change
1	cg00574958	11	68,607,622	*CPT1A*	-18.22	2.79	0.10 ± 0.01	1.36 × 10^− 10^	4.21 × 10^− 7^	-8.044 [-10.458–-5.631]
2	cg17901584	1	55,353,706	*DHCR24*	-6.97	1.08	0.42 ± 0.05	2.26 × 10^− 10^	4.21 × 10^− 7^	-3.078 [-4.013–-2.143]
3	cg17061862	11	9,590,431	NA	-5.42	0.96	0.60 ± 0.04	2.38 × 10^− 8^	2.95 × 10^− 5^	-2.393 [-3.222–-1.563]
4	cg01787285	1	2,162,682	*SKI*	-7.35	1.39	0.17 ± 0.03	1.65 × 10^− 7^	0.0002	-3.244 [-4.444–-2.043]
5	cg19750657	13	38,935,967	*UFM1*	6.71	1.37	0.64 ± 0.04	1.34 × 10^− 6^	0.001	2.963 [1.773–4.152]
6	cg17058475	11	68,607,737	*CPT1A*	-9.28	2.13	0.10 ± 0.02	1.54 × 10^− 5^	0.01	-4.095 [-5.936–-2.254]
7	cg19693031	1	145,441,552	*TXNIP*	-3.87	0.90	0.64 ± 0.05	1.92 × 10^− 5^	0.01	-1.708 [-2.485–-0.931]
8	cg01176028	21	43,653,234	*ABCG1*	8.16	1.98	0.21 ± 0.03	4.39 × 10^− 5^	0.02	3.604 [1.887–5.320]
9	cg04524040	19	4,153,364	*CREB3L3*	-5.66	1.39	0.38 ± 0.04	4.96 × 10^− 5^	0.02	-2.500 [-3.699–-1.301]
10	cg05901543	16	89,251,975	*CDH15*	3.54	0.88	0.64 ± 0.05	6.27 × 10^− 5^	0.02	1.564 [0.804–2.325]

BMI, measured as inverse-normal BMI residuals after regressing on age and sex, was treated as the outcome, with individual CpGs as independent variables in the linear regression analysis. The results were additionally adjusted for smoking status, age, and the first ten Principal Components (PCs). BMI change is defined as the change in raw BMI (kg/m²) per 10% (i.e., 0.1 unit) increase in the methylation beta value, assuming smoking status and other covariates are held constant, with confidence intervals CI provided in brackets.

### Polygenic risk score and sensitivity analysis

**Fig. 3:**
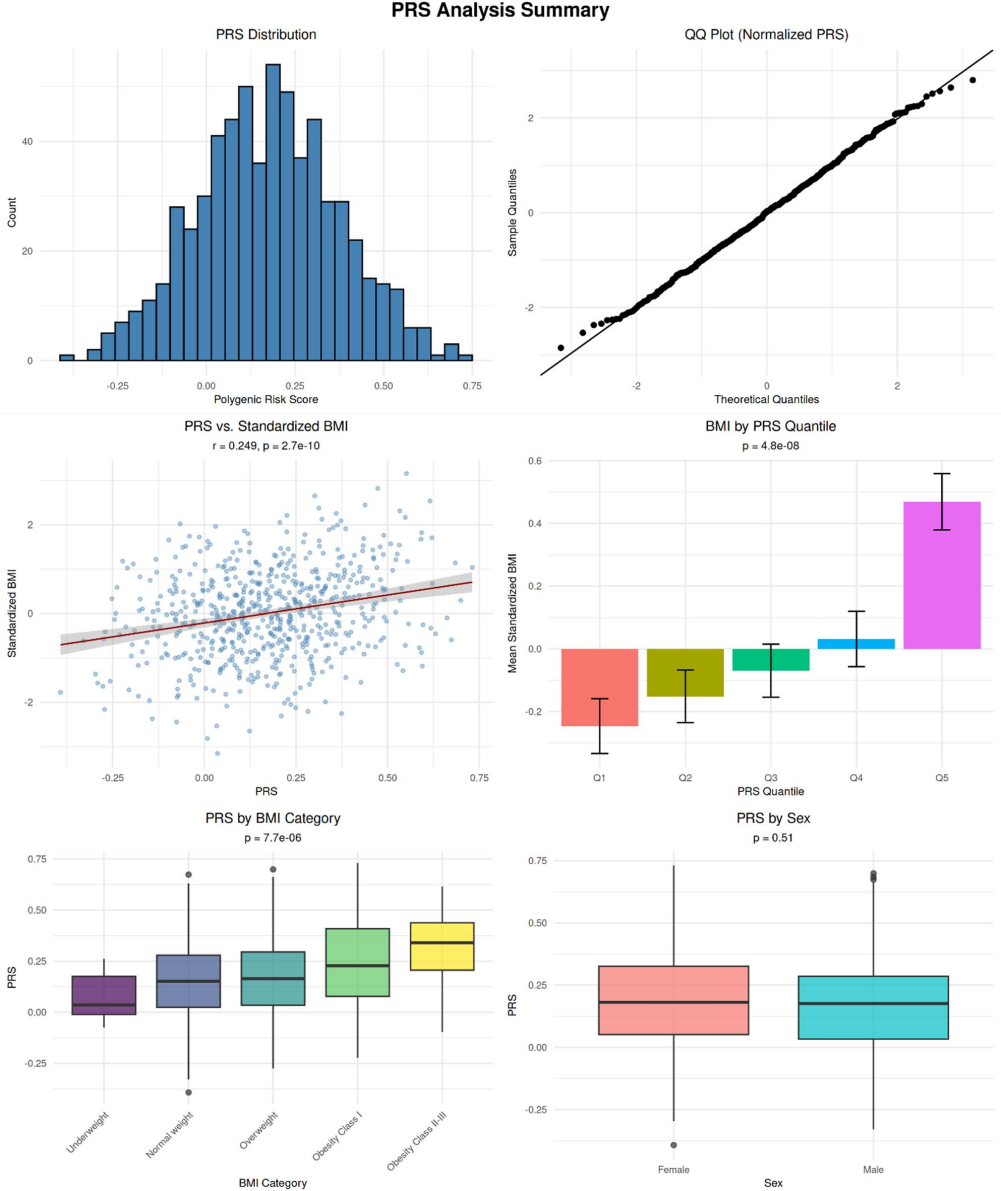
Polygenic risk score (PRS) analysis based on GWAS summary statistics from Yengo et al., 2018

The polygenic risk score was calculated for a main sample set of *N* = 624 using GWAS summary statistics from Yengo et al. [32]. The distribution of PRS values and between-group comparisons are shown in Fig. 3. In our cohort, PRS showed a weak-to-moderate, but highly significant correlation with BMI, (*r* = 0.249, *p* = 2.7 × 10^− 10^). When phenotypic BMI was categorized into underweight (BMI < 18.5), normal weight (BMI 18.5–24.9), overweight (BMI 25.0–29.9), obesity class I (BMI 30.0–34.9), and obesity class II–III (BMI > 35), the comparison also reached statistical significance (*p* = 7.7 × 10^− 6^). No significant differences in PRS were observed between females and males (*p* = 0.51). The PRS for overweight status was additionally calculated [40, 41] and showed a lower, yet still significant, correlation with BMI in our cohort (*r* = 0.104, *p* = 0.009; **Fig. S2**).

Finally, the generated polygenic risk scores from Yengo et al. were used in a sensitivity analysis to assess whether the association between DNA methylation and BMI was confounded by genetic variants and the effect was negligible (**Table S4**).

### Comparative analysis of epigenetic predictors of BMI

**Fig. 4:**
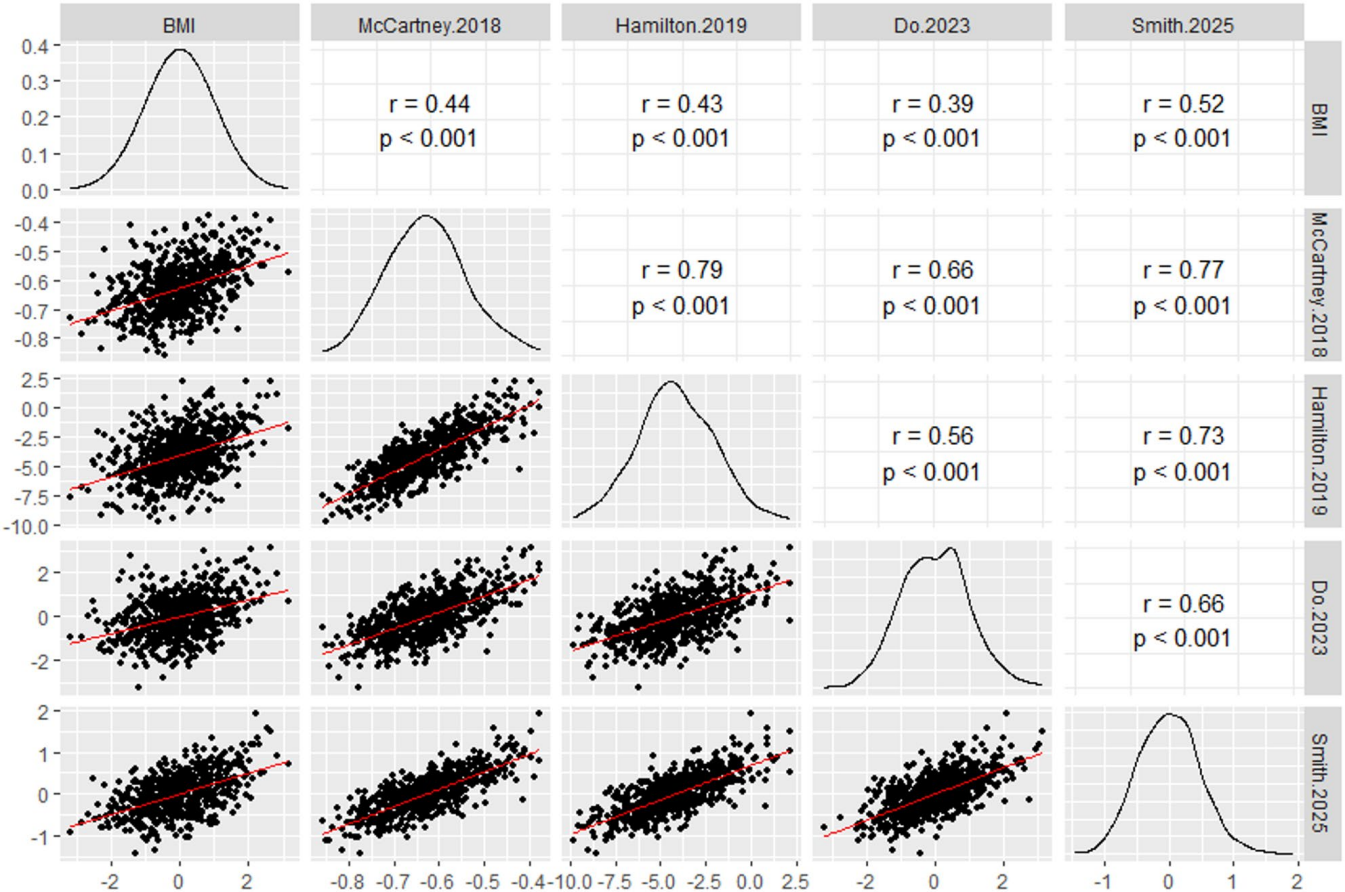
Correlation matrix between different epigenetic BMI predictors and phenotypic BMI. BMI is presented as inverse-normal BMI residuals

Four published epigenetic predictors of BMI were evaluated for their correlation with phenotypic BMI in our study sample (Set 1 *N* = 624). The number of cytosines analyzed varied, ranging from approximately 400 to nearly 2,000 [5, 11, 14, 15]. All models demonstrated a significant moderate correlation with BMI, with correlation coefficients ranging from 0.39 to 0.52 (Fig. 4). The strongest effect (*r* = 0.52, *p* = 1.7 × 10^− 44^) was observed for the most recent and most CpG-dense predictor, developed by Smith et al. (2025) [5]. Across the models, the output of the Hamilton (2019) predictor [14] showed a strong correlation with that of McCartney (2018) [11] (*r* = 0.79, *p* = 1.1 × 10^− 133^), and the McCartney (2018) predictor, in turn, correlated strongly with the Smith (2025) predictor [5] (*r* = 0.77, *p* = 5.5 × 10^− 125^). All comparisons yielded correlation coefficients of 0.56 or higher (Fig. 4).

**Fig. 5:**
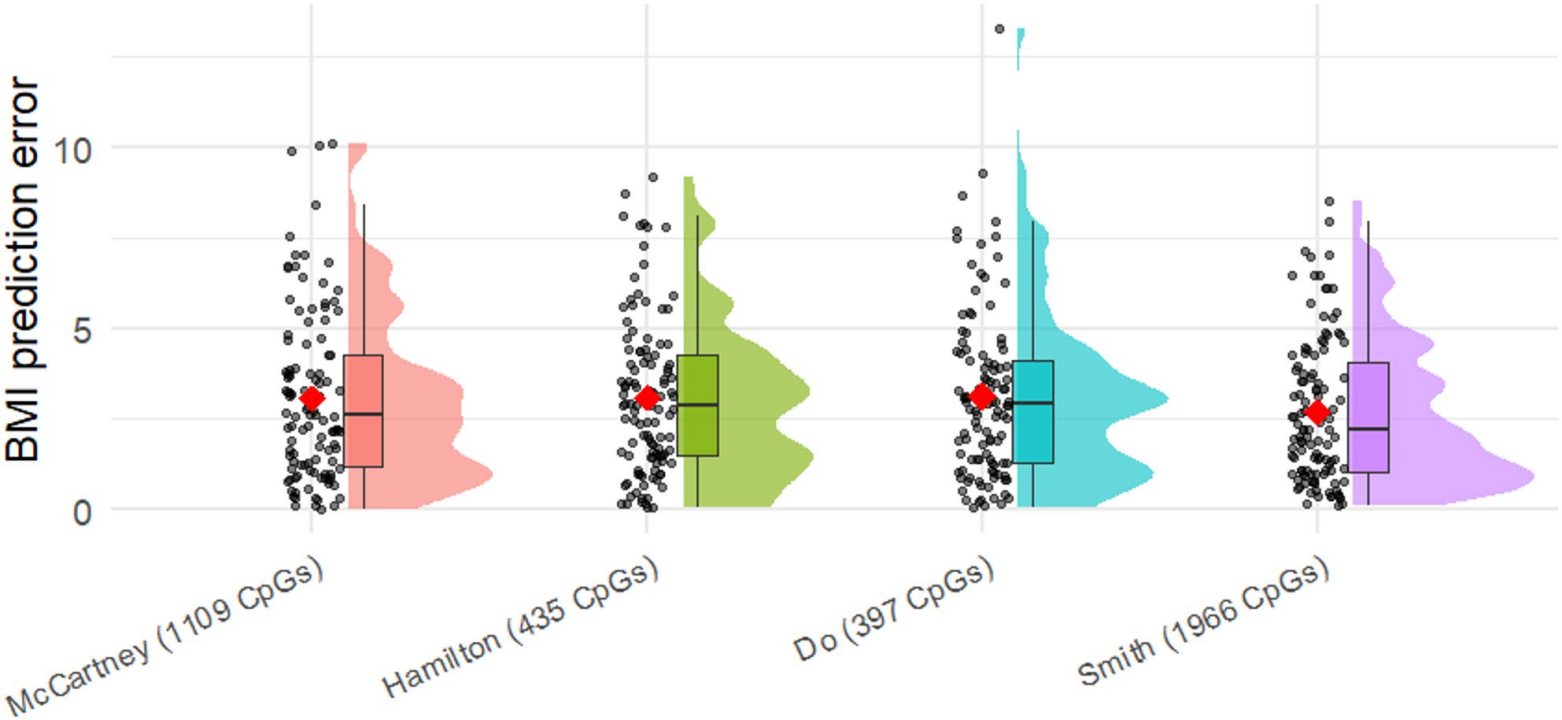
Raincloud plot illustrating BMI prediction error across various predictors. The plot compares absolute prediction errors, defined as the differences between predicted and phenotypic BMI values. The horizontal line within each distribution denotes the median error, while the red dot represents the mean absolute error (MAE), which was 3.07, 3.11, 3.14, 2.71 for the McCartney, Hamilton, Do, Smith models, respectively. The number of predictors used in each model is indicated in parentheses. The analysis was conducted on the independent test set (Set 2 N = 112). As the McCartney, Hamilton, Do, and Smith models do not provide direct estimates of absolute BMI, a linear regression model was fitted on the main set (Set 1 N = 624) beforehand to map predicted values to phenotypic BMI, and this transformation was then applied to the Set 2.

As the outputs of the applied models represent scaled rather than absolute BMI values, a linear regression model was fitted to a dataset of *N* = 624 samples to map the predicted values to absolute phenotypic BMI. This transformation was subsequently applied to an independent test set of *N* = 112 samples, and the mean absolute error (MAE) of BMI predictions was evaluated (Fig. 5). The model by Smith et al. demonstrated the highest accuracy, with an MAE of 2.71. The remaining three models predicted BMI with an accuracy of approximately ± 3.1 BMI units.

### Linking BMI prediction with epigenetic health markers

**Fig. 6:**
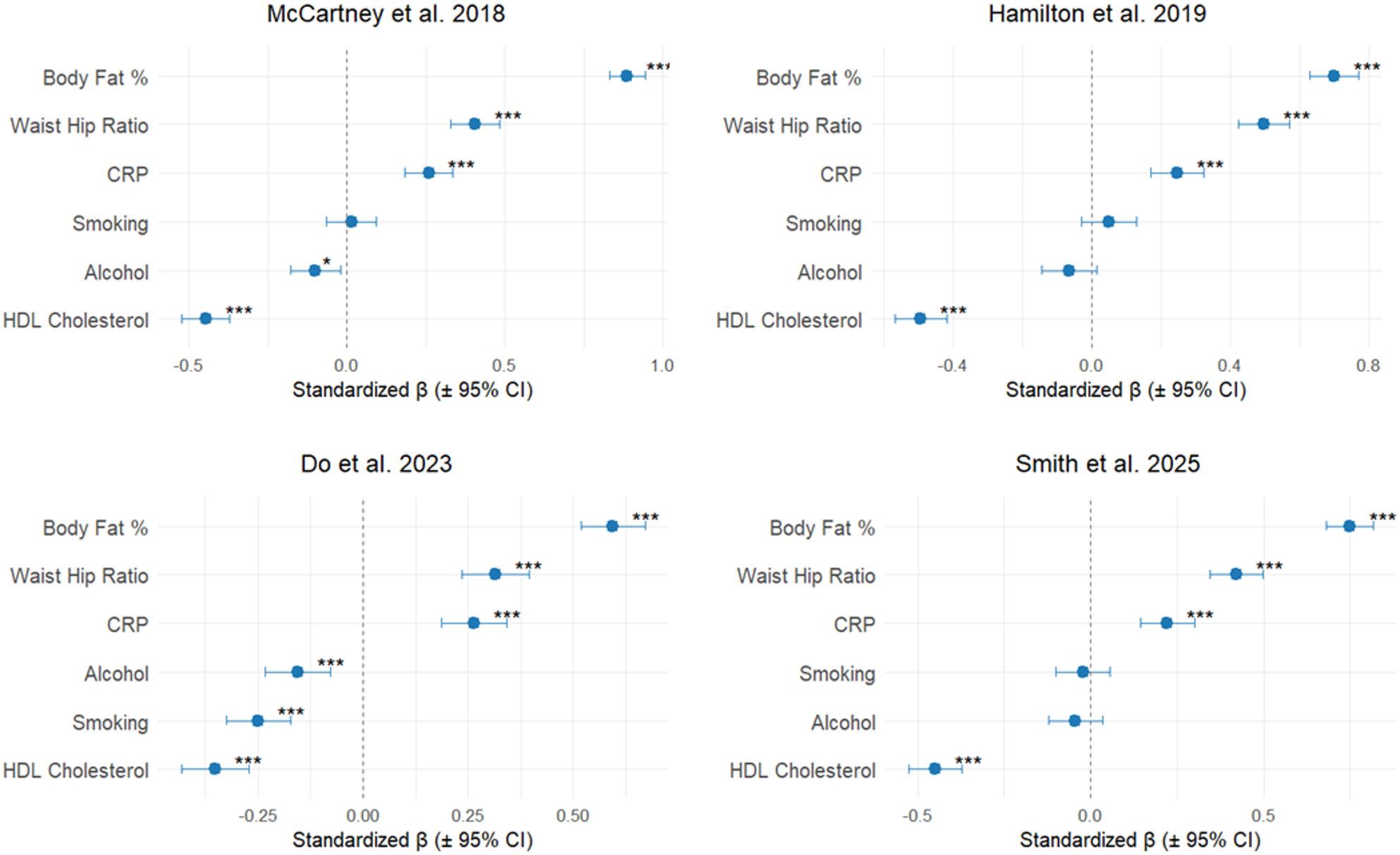
Forest plots illustrating the strength of associations between epigenetic BMI and a set of established epigenetic proxies of health metrics from Marioni et al. [44] and Ligthart et al. [45]. The analysis tested associations between these proxies and the residuals from the regression of epigenetic BMI (predicted using each model) on phenotypic BMI, adjusted for sex and age. These residuals represent the component of epigenetic BMI not explained by phenotypic BMI. Asterisks indicate levels of statistical significance: *p* ≤ 0.05 (*), *p* ≤ 0.01 (**), *p* ≤ 0.001 (***)

Finally, BMI predicted using various (epi)genetic models was evaluated for its potential correlation with metabolic health, and it was assessed whether these associations were independent of phenotypic BMI. To this end, residuals from the regression of epigenetic BMI (predicted using each model) on phenotypic BMI were tested for associations with various epigenetic health markers. Among the evaluated models, the epigenetic BMI model published by Do et al. [15] showed the most consistent associations between BMI prediction residuals and various epigenetic health measures (all *p* < 0.001; Fig. 6). Across all models, the strongest effect size of association, measured by the standardized beta coefficient, was observed for body fat percentage (β ≥ 0.6). Waist-to-hip ratio and CRP were the second and third most strongly associated measures. Smoking was associated only with the Do et al. model, while alcohol consumption was significant for both the McCartney [11] and Do [15].

## Discussion

Body mass index (BMI) is a commonly used measure of obesity and mounting evidence suggests that both genetic variation and epigenetic modifications play key roles in regulating body weight and adiposity-related pathways. Numerous genetic variants and DNA methylation loci associated with BMI have been reported, prompting the development of polygenic and epigenetic scores [5, 11, 14, 15, 32], with the latter capturing environmental exposures and habits, thus offering a broader window into the molecular landscape of obesity. Building on these advances, we validated existing (epi)genetic BMI models in an independent population and performed the first comprehensive comparison of these tools.

The polygenic risk score reported by Yengo et al. exhibited only a low correlation with phenotypic BMI (r ∼ 0.22) and explained only 6% of its variance in the original study [32]. Projection of this PRS onto our dataset revealed a similar correlation (*r* = 0.249). Furthermore, only 4.9% of the 371 SNPs selected as candidates in our study and present on the GSA microarray showed nominally significant associations with BMI. Among the top 10 significant SNPs, rs10968576 stands out as the most robustly validated marker, having been associated with BMI in at least three large studies [30, 31, 33]. It is located in the *LINGO2* gene, which encodes the leucine rich repeat and Ig domain containing protein 2, primarily implicated in various neurological disorders and cancer development [47], and has been associated in some studies with gestational diabetes [48, 49]; however, its molecular function in metabolic homeostasis remains largely unexplored. The most significant variant in our study, rs11688816, is located within the *EHBP1* gene, which is involved in the intracellular trafficking of GLUT4, a glucose transporter essential for insulin-stimulated glucose uptake in adipose tissue [50]. Position rs4776970, the second most significant SNP is located in the *MAP2K5* gene, which regulates adipogenesis through the ERK5 signaling pathway and contributes to fat cell differentiation [51]. Interestingly, rs9400239 at *FOXO3* was associated with BMI in our study. Since this gene is one of the most well-known longevity genes [52], this finding highlights a potential link between obesity and aging.

Analysis of epigenetic modifications may add to the variation observed in BMI by capturing the impact of environmental exposures, an important contributor to obesity risk. Notably, Mendelian Randomization analyses have suggested that alterations in DNA methylation may play a causal role, with specific CpG sites reported to impact BMI regulation [18]. In recent years, numerous BMI-associated DNA methylation markers have been identified, with current epigenetic BMI scores relying on hundreds of CpG sites [5, 11, 14, 15]. Interestingly, the overlap of CpG sites between the epigenetic BMI classifiers is minimal, as shown in the Venn diagram (Fig. 1). This observation may suggest that the impact of BMI on DNA methylation can span a wide range of CpG sites. However, differences in the sets of predictors may also arise from technical variation, including differences in array platforms, preprocessing pipelines, normalization methods, and feature-selection strategies. The lower heritability of BMI than for many other physical traits [53], does not allow for much optimism about genetic prediction of this trait. Consequently, the most reliable BMI predictors reported in the literature are based on DNA methylation markers. In our study, we successfully replicated 8.6% of the known BMI-associated CpGs, with associations significantly stronger than those observed for SNPs. The two top-performing CpGs (raw *p* < 10⁻^9^), were cg00574958 in *CPT1A* and cg17901584 in *DHCR24*. Their associations with BMI have been consistently reported across multiple studies [5, 11, 14–18], both featuring in all four established BMI epigenetic models [5, 11, 14, 15]. The *CPT1A* gene encodes a key enzyme responsible for transporting long-chain fatty acids into mitochondria, where they are oxidized to produce energy [54]. Maintaining energy balance is crucial to prevent excess fat storage and disruptions of this process may contribute to obesity and associated metabolic disorders [55]. *DHCR24* encodes an enzyme essential for the final step of cholesterol biosynthesis, converting 24-dehydrocholesterol into cholesterol [56, 57]. Its role in sterol metabolism and lipid homeostasis may influence fat storage and overall energy balance. *DHCR24* also has antioxidant and antiapoptotic functions that help protect cells, including adipocytes, from oxidative stress, a process increased in obesity and linked to metabolic dysfunction [58]. A very recent study reported a significant correlation between BMI and *DHCR24* expression levels [57].

Predicting BMI from (epi)genetic data may be valuable in fields such as forensics and anthropology by providing additional information about individuals under investigation. These analyses fall within the discipline of Forensic DNA Phenotyping (FDP), applied in cases lacking suspects where standard comparative analysis based on STR markers cannot be applied.

FDP seeks to extract a range of information from DNA, including sex, age, biogeographical ancestry, physical appearance, and behavioral traits, enabling the reconstruction of a suspect’s profile, narrowing down the suspect pool, and accelerating the investigation process [22, 24, 59]. BMI contributes to variation in facial features and could therefore enhance predictive analyses that are currently undertaken using genetic variants and sex [25, 60].

Predicting BMI is also explored in diagnosing metabolic diseases. Obesity stands as one of the leading public health concerns, predisposes to chronic diseases, including type 2 diabetes, cardiovascular disease, myocardial infarction, and stroke, and is also associated with certain cancers, impaired cognitive function, and reduced life expectancy [2, 4, 61–65]. BMI, calculated from weight and height, is one of the most widely used indicators of obesity, yet it captures only a limited view of metabolic health. In particular, it has limited capacity to accurately reflect total body fat or its distribution, often failing to identify individuals with excess adiposity despite a normal BMI [3, 6, 66]. In contrast, predicting BMI from genetic or epigenetic markers may offer a way to uncover biological pathways underlying adiposity and, in turn, provide a more accurate understanding of associated health outcomes [5]. For example, Hamilton et al. demonstrated that utilizing an epigenetic BMI score increased the explained variance in metabolic risk factors beyond that attributed to phenotypic BMI alone by several percentage points [14]. The superiority of the epigenetic counterpart to the conventional immunochemistry-based measure has also been demonstrated in the literature, for example, for CRP [67].

Epigenetic BMI models examined in our study, were originally reported to explain between 10% and 20% of the variance in phenotypic BMI [5, 11, 14, 15]. In our cohort, the top ten CpG sites accounted for 15.5% of the variance, while the tested models showed moderate correlations with BMI, ranging from 0.39 to 0.52. The strongest correlation was observed for the recently published Smith model [5]. The majority of existing studies do not focus primarily on the prediction of BMI itself, but rather on investigating associations between epigenetic BMI signatures and various health-related outcomes. In the study by Llobet et al. (2023), prediction errors ranged from 3.8 to 5.8 BMI units when using DNA methylation data alone [68]. Notably, the authors demonstrated that integrating methylation data with plasma protein profiles, such as adiponectin and leptin, which are closely linked to body composition, substantially improved prediction accuracy, reducing the error to 2.8–3.3 BMI units. In our comparative analysis, the mean absolute error (MAE) was approximately 3.1 BMI units across the models, but it fell below this threshold for the most recent model proposed by Smith et al. [5]. However, it should be noted that existing epigenetic models in the literature do not predict absolute BMI values. Therefore, to enable an in-depth analysis, we applied a linear transformation to map epigenetic scores, which represent relative BMI values, onto phenotypic BMI values using our population data, as suggested by Smith et al. [5]. This step, however, may have introduced a source of error in the prediction. McCartney et al. employed categorical BMI classification and an AUC of 0.71 was reported for distinguishing obese from non-obese individuals, although the model incorporated a polygenic risk score [11]. The highest AUC reported to date (0.791) was achieved in a meta-analysis by Do et al., in which BMI was predicted from DNA methylation at 397 CpG sites [15].

Finally, we examined the link between discrepancies in predicted versus phenotypic BMI and various epigenetic health markers. We observed a particularly strong association with body fat percentage, supporting the hypothesis that epigenetic BMI may serve as a more precise indicator of body fat composition than phenotypic BMI [6, 66]. Deviations from phenotypic BMI were also frequently associated with epigenetically inferred waist-to-hip ratio and CRP levels across various published models, suggesting a potential link between inflammation and metabolic dysfunction [69, 70]. However, as a limitation of our study, it is important to note that the association analyses were performed using epigenetic proxies rather than direct measurements of specific health metrics, and the potential confounding effects of underlying CpG sites should be considered.

Finally, it should be recognized that current predictors represent only a first step toward the efficient implementation of BMI‑prediction models. Since PRS models are typically based on SNP data from microarray platforms, which cover rare variants only to a very limited extent, they may not fully account for the phenotypic variance in BMI attributable to high‑effect monogenic variants. In particular, loss-of-function mutations in genes of the leptin–melanocortin pathway have been shown to contribute to severe obesity risk [71–73]. While these monogenic variants are relatively rare in the general population, large population-based analyses (e.g., UK Biobank) have demonstrated that heterozygous carriers of rare loss‑of‑function variants in *MC4R*, *POMC*, and *PCSK1* show higher BMI compared to non-carriers [73]. Furthermore, the penetrance of obesity among carriers in these studies ranged broadly (median ~ 23%), suggesting that not all carriers develop obesity, and that polygenic background may modulate phenotypic expression. Future efforts could integrate rare-variant data (e.g., from exome or whole-genome sequencing) to enhance both the accuracy and interpretability of predictive models, especially in individuals with extreme or early-onset obesity.

## Conclusions

In this study, we compared large epigenetic and genetic BMI prediction models and evaluated their correlation with phenotypic BMI, as well as with other epigenetic scores related to metabolic health. Our study integrated two data types: DNA methylation and SNPs. While SNPs contributed to the explained variance, CpG markers emerged as the primary drivers, showing markedly stronger effect sizes in the studied population. We found that the most recent model by Smith et al., which was trained on the largest number of CpG sites, showed the strongest correlation with BMI. In contrast, the model by Do et al., developed from a large meta-analysis, exhibited the most robust associations across various epigenetic markers, including body fat percentage, CRP, smoking, and alcohol consumption. While our results indicate that these tools may have clinical relevance within the studied population, further research is necessary to assess their utility in predicting metabolic disorder risk, especially among individuals with a normal phenotypic BMI. Conversely, in forensic contexts, our findings may guide the selection of models with the lowest prediction error. However, the constraints of limited and degraded samples suggest that developing a streamlined model tailored to forensic needs could be beneficial in future studies. Moreover, the potential of epigenetic BMI to enhance appearance reconstruction still requires thorough evaluation. Finally, given the technical and biological sources of variability that cannot be fully excluded, the findings of our study should be interpreted as exploratory and warrant independent replication.

## Supplementary Information

Below is the link to the electronic supplementary material.

Supplementary Material 1

## Data Availability

The raw microarray data supporting the findings of this study have been deposited in a public repository and are available through controlled access at RODBUK 10.57903/UJ/Q6SA9L.
